# *CYP19A1* fine-mapping and Mendelian randomization: estradiol is causal for endometrial cancer

**DOI:** 10.1530/ERC-15-0386

**Published:** 2016-02

**Authors:** Deborah J Thompson, Tracy A O'Mara, Dylan M Glubb, Jodie N Painter, Timothy Cheng, Elizabeth Folkerd, Deborah Doody, Joe Dennis, Penelope M Webb, for the Australian National Endometrial Cancer Study Group (ANECS), Maggie Gorman, Lynn Martin, Shirley Hodgson, for the National Study of Endometrial Cancer Genetics Group (NSECG), Kyriaki Michailidou, Jonathan P Tyrer, Mel J Maranian, Per Hall, Kamila Czene, Hatef Darabi, Jingmei Li, Peter A Fasching, Alexander Hein, Matthias W Beckmann, Arif B Ekici, Thilo Dörk, Peter Hillemanns, Matthias Dürst, Ingo Runnebaum, Hui Zhao, Jeroen Depreeuw, Stefanie Schrauwen, Frederic Amant, Ellen L Goode, Brooke L Fridley, Sean C Dowdy, Stacey J Winham, Helga B Salvesen, Jone Trovik, Tormund S Njolstad, Henrica M J Werner, Katie Ashton, Tony Proietto, Geoffrey Otton, Luis Carvajal-Carmona, Emma Tham, Tao Liu, Miriam Mints, for RENDOCAS, Rodney J Scott, Mark McEvoy, John Attia, Elizabeth G Holliday, Grant W Montgomery, Nicholas G Martin, Dale R Nyholt, Anjali K Henders, John L Hopper, Nadia Traficante, for the AOCS Group, Matthias Ruebner, Anthony J Swerdlow, Barbara Burwinkel, Hermann Brenner, Alfons Meindl, Hiltrud Brauch, Annika Lindblom, Diether Lambrechts, Jenny Chang-Claude, Fergus J Couch, Graham G Giles, Vessela N Kristensen, Angela Cox, Manjeet K Bolla, Qin Wang, Stig E Bojesen, Mitul Shah, Robert Luben, Kay-Tee Khaw, Paul D P Pharoah, Alison M Dunning, Ian Tomlinson, Mitch Dowsett, Douglas F Easton, Amanda B Spurdle

**Affiliations:** 1 Department of Public Health and Primary Care, Centre for Cancer Genetic Epidemiology, University of Cambridge, Strangeways Research Laboratory, Worts Causeway, Cambridge, CB1 8RN, UK; 2 Department of Genetics and Computational Biology, QIMR Berghofer Medical Research Institute, Brisbane, Queensland, 4006, Australia; 3 Wellcome Trust Centre for Human Genetics, University of Oxford, Oxford, OX3 7BN, UK; 4 Academic Department of Biochemistry, Royal Marsden Hospital, London, SW3 6JJ, UK; 5 Department of Clinical Genetics, St George's Hospital Medical School, London, SW17 0RE, UK; 6 Department of Oncology, Centre for Cancer Genetic Epidemiology, University of Cambridge, Cambridge, CB1 8RN, UK; 7 Department of Medical Epidemiology and Biostatistics, Karolinska Institutet, Stockholm, SE-171 77, Sweden; 8 Department of Medicine, Division of Hematology/Oncology, David Geffen School of Medicine, University of California at Los Angeles, Los Angeles, California, 90095, USA; 9 Department of Gynecology and Obstetrics, University Hospital Erlangen, Friedrich-Alexander-University Erlangen-Nuremberg, Erlangen, 91054, Germany; 10 Institute of Human Genetics, , University Hospital Erlangen, Friedrich-Alexander-University Erlangen-Nuremberg, Erlangen, 91054, Germany; 11 Gynaecology Research Unit, Hannover Medical School, Hannover, 30625, Germany; 12 Clinics of Gynaecology and Obstetrics, Hannover Medical School, Hannover, 30625, Germany; 13 Department of Gynaecology, Jena University Hospital – Friedrich Schiller University, Jena, 07743, Germany; 14 Vesalius Research Center, Leuven, 3000, Belgium; 15 Laboratory for Translational Genetics, Department of Oncology, University Hospitals Leuven, Leuven, 3000, Belgium; 16 Department of Obstetrics and Gynecology, Division of Gynecologic Oncology, University Hospitals, KU Leuven – University of Leuven, Leuven, 3000, Belgium; 17 Department of Health Sciences Research, Mayo Clinic, Rochester, Minnesota, 55905, USA; 18 Department of Biostatistics, University of Kansas Medical Center, Kansas City, Kansas, 66160, USA; 19 Department of Obstetrics and Gynecology Division of Gynecologic Oncology Mayo Clinic, Rochester, Minnesota, 55905, USA; 20 Department of Clinical Science, Centre for Cancerbiomarkers, The University of Bergen, Bergen, 5020, Norway; 21 Department of Obstetrics and Gynecology, Haukeland University Hospital, Bergen, 5021, Norway; 22 Hunter Medical Research Institute, John Hunter Hospital, Newcastle, New South Wales, 2305, Australia; 23 Centre for Information Based Medicine, University of Newcastle, Newcastle, New South Wales, 2308, Australia; 24 School of Biomedical Sciences and Pharmacy, , University of Newcastle Newcastle, Newcastle, New South Wales, 2308, Australia; 25 School of Medicine and Public Health, , University of Newcastle, Newcastle, Newcastle, New South Wales, 2308, Australia; 26 Grupo de investigación Citogenética, Filogenia y Evolución de Poblaciones, Universidad del Tolima, Ibagué, Tolima, Colombia; 27 Genome Center and Department of Biochemistry and Molecular Medicine, University of California, Davis, California, 95616, USA; 28 Department of Molecular Medicine and Surgery, Karolinska Institutet, Stockholm, SE-171 77, Sweden; 29 Department of Women's and Children's Health, Karolinska Institutet, Karolinska University Hospital, Stockholm, SE-171 77, Sweden; 30 Hunter Area Pathology Service, John Hunter Hospital, Newcastle, New South Wales, 2305, Australia; 31 Centre for Clinical Epidemiology and Biostatistics, School of Medicine and Public Health, University of Newcastle, Newcastle, New South Wales, 2305, Australia; 32 Institute of Health and Biomedical Innovation, Queensland University of Technology, Brisbane, 4006, Australia; 33 Centre for Epidemiology and Biostatistics, Melbourne School of Population and Global Health, The University of Melbourne victoria, Melbourne, Victoria, 3010, Australia; 34 PePeter MacCallum Cancer Center, The University of Melbourne, Melbourne, 3002, Australia; 35 Division of Genetics and Epidemiology, Institute of Cancer Research, London, SM2 5NG, UK; 36 Division of Breast Cancer Research, Institute of Cancer Research, London, SM2 5NG, UK; 37 Department of Gynecology and Obstetrics, Molecular Biology of Breast Cancer, University of Heidelberg, Heidelberg, 69117, Germany; 38 Division of Cancer Epidemiology, German Cancer Research Center, Heidelberg, 69120, Germany; 39 Division of Clinical Epidemiology and Aging Research, German Cancer Research Center (DKFZ), Heidelberg, 69120, Germany; 40 Division of Preventive Oncology, German Cancer Research Center (DKFZ), Heidelberg, 69120, Germany; 41 German Cancer Consortium (DKTK) and German Cancer Research Center (DKFZ), Heidelberg, 69120, Germany; 42 Department of Obstetrics and Gynecology, Division of Tumor Genetics, Technical University of Munich, Munich, 80333, Germany; 43 Dr Margarete Fischer-Bosch-Institute of Clinical Pharmacology, Stuttgart, 70376, Germany; 44 University of Tübingen, Tübingen, 72074, Germany; 45 Department of Laboratory Medicine and Pathology, Mayo Clinic, Rochester, Minnesota, 55905, USA; 46 Cancer Epidemiology Centre, Cancer Council Victoria, Melbourne, Victoria, 3004, Australia; 47 Department of Epidemiology and Preventive Medicine, Monash University, Melbourne, Victoria, 3004, Australia; 48 Department of Genetics, Institute for Cancer Research, The Norwegian Radium Hospital, Oslo, 0310, Norway; 49 Faculty of Medicine, The K.G. Jebsen Center for Breast Cancer Research, Institute for Clinical Medicine, , University of Oslo, Oslo, 0316, Norway; 50 Department of Clinical Molecular Oncology, Division of Medicine, Akershus University Hospital, Lørenskog, 1478, Norway; 51 Department of Oncology, Sheffield Cancer Research, University of Sheffield, Sheffield, S10 2TN, UK; 52 Faculty of Health and Medical Sciences, University of Copenhagen, Copenhagen, 1165, Denmark; 53 Department of Clinical Biochemistry, Herlev Hospital, Copenhagen University Hospital, Herlev, 2730, Denmark; 54 Department of Public Health and Primary Care, University of Cambridge, Cambridge, CB1 8RN, UK; 55 MRC Centre for Nutritional Epidemiology in Cancer Prevention and Survival (CNC), University of Cambridge, Cambridge, CB1 8RN, UK

**Keywords:** endometrial cancer, CYP19A1, estradiol

## Abstract

Candidate gene studies have reported *CYP19A1* variants to be associated with endometrial cancer and with estradiol (E_2_) concentrations. We analyzed 2937 single nucleotide polymorphisms (SNPs) in 6608 endometrial cancer cases and 37 925 controls and report the first genome wide-significant association between endometrial cancer and a *CYP19A1* SNP (rs727479 in intron 2, *P*=4.8×10^−11^). SNP rs727479 was also among those most strongly associated with circulating E_2_ concentrations in 2767 post-menopausal controls (*P*=7.4×10^−8^). The observed endometrial cancer odds ratio per rs727479 A-allele (1.15, CI=1.11–1.21) is compatible with that predicted by the observed effect on E_2_ concentrations (1.09, CI=1.03–1.21), consistent with the hypothesis that endometrial cancer risk is driven by E_2_. From 28 candidate-causal SNPs, 12 co-located with three putative gene-regulatory elements and their risk alleles associated with higher *CYP19A1* expression in bioinformatical analyses. For both phenotypes, the associations with rs727479 were stronger among women with a higher BMI (*P*_interaction_=0.034 and 0.066 respectively), suggesting a biologically plausible gene-environment interaction.

## Introduction

Established risk factors for endometrial cancer include high BMI (IARC 2002), early menarche, late menopause, nulliparity, estrogen-only hormone replacement therapy (HRT) (Beral *et al*. 2005) and tamoxifen use (Cuzick *et al*. 2003), while cigarette smoking and the use of oral contraceptives or combined HRT (Beral *et al*. 2005) are associated with lower risks. It has been hypothesized that these factors alter endometrial cancer risk by increasing exposure to estrogens (Key & Pike 1988); indeed, higher concentrations of circulating estradiol (E_2_) in post-menopausal women have been associated with an increased risk of endometrial cancer (Zeleniuch-Jacquotte *et al*. 2001, Lukanova *et al*. 2004, Allen *et al*. 2008).

After the cessation of ovarian estrogen production at menopause, endogenous estrogens are primarily synthesized from testosterone (T) in adipose tissue via aromatase, encoded by *CYP19A1*. Candidate gene studies have found levels of E_2_ in pre-menopausal and post-menopausal women, and also in men, to be associated with genetic variants within or close to *CYP19A1* (Dunning *et al*. 2004, Paynter *et al*. 2005, Haiman *et al*. 2007, Ahn *et al*. 2009, Eriksson *et al*. 2009, Kidokoro *et al*. 2009, Travis *et al*. 2009, Beckmann *et al*. 2011, Lundin *et al*. 2012, Prescott *et al*. 2012, Flote *et al*. 2014). Candidate studies have also identified associations between several different *CYP19A1* variants and endometrial cancer (Paynter *et al*. 2005, Tao *et al*. 2007, Setiawan *et al*. 2009, Low *et al*. 2010), with some evidence of stronger associations in women with higher BMI (Setiawan *et al*. 2009).

**Figure 1:**
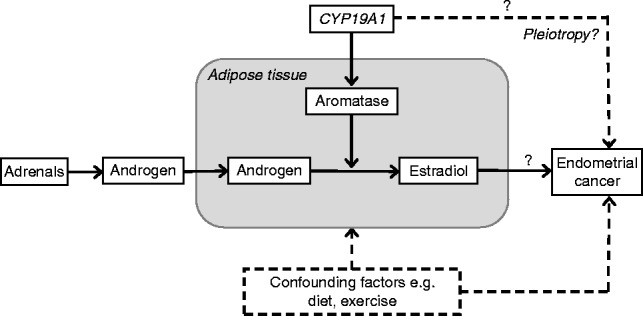
Factors potentially involved in the reported association between circulating post-menopausal E_2_ and endometrial cancer risk. Question marks highlight the issues to be addressed in this study.

None of the published studies have attempted a systematic assessment of all common *CYP19A1* variants in order to determine i) which are most likely to be causal for endometrial cancer and/or E_2_ concentration, ii) whether multiple independent causal variants exist at this locus for either trait, and iii) whether the same variant or variants are responsible for both traits. The latter would help to address the question as to whether the reported association between E_2_ and endometrial cancer seen in epidemiological studies is causal or a consequence of confounding (Fig. 1). If the association is causal, then variants causally associated with E_2_ levels should also be associated with endometrial cancer, with a magnitude that can be predicted using a Mendelian randomization methodology (C-Reactive Protein Coronary Heart Disease Genetics Collaboration *et al*. 2011, Interleukin-6 Receptor Mendelian Randomisation Analysis Consortium 2012), a form of instrumental variable analysis in which the instrument is a genetic variant(s) known to be associated with the biomarker in a particular direction.

To address the question of whether the same *CYP19A1* variant(s) are associated with E_2_ levels and endometrial cancer with compatible effect sizes and directions, we used genotype information for 2937 single nucleotide polymorphisms (SNPs) across a 1.2 Mb region encompassing *CYP19A1* in 6608 endometrial cancer cases and 37 925 controls of European ancestry, 1733 of whom (all controls) were post-menopausal and had measured E_2_ and T concentrations.

## Materials and methods

### Endometrial cancer case–control studies

The association between SNPs at the *CYP19A1* locus and endometrial cancer was tested using data from four separate case-control studies:

#### The ANECS, SEARCH, and NSECG genomewide association studies

The results presented here are based on the ANECS, SEARCH, and NSECG genomewide association studies (GWAS) and country-matched datasets (McGregor *et al*. 1999, WTCCC 2007, Houlston *et al*. 2010, McEvoy *et al*. 2010, Painter *et al*. 2011, Spurdle *et al*. 2011), as shown in Supplementary Table 1A and B, see section on supplementary data given at the end of this article, and described in detail in (Painter *et al*. 2015). All cases and controls were of European ancestry.

#### The ECAC iCOGS study

The fourth dataset comprised 4402 women of European ancestry with a confirmed diagnosis of endometrial cancer (3535 with confirmed endometrioid histology), recruited via 11 separate studies in seven countries in the Endometrial Cancer Association Consortium (ECAC; Painter *et al*. 2015) and 28 758 healthy female controls from the same countries, all participating in the Breast Cancer Association Consortium (BCAC; Michailidou *et al*. 2013) or the Ovarian Cancer Association Consortium (OCAC; Pharoah *et al*. 2013), plus 282 Norwegian blood donor controls (see Supplementary Information, Supplementary Table 1A and B, see section on supplementary data given at the end of this article). Cases and controls were genotyped for 211 155 SNPs using a custom Illumina Infinium iSelect array (‘iCOGS’; Michailidou *et al*. 2013, Pharoah *et al*. 2013, Painter *et al*. 2015) designed by the Collaborative Oncological Gene-environment Study (‘COGS’). The iCOGS array includes 134 SNPs located within the ∼1.2 Mb region of chromosome 15 between 50 899 000–52 095 000 surrounding *CYP19A1*, 22 of which had been specifically selected for the study of post-menopausal E_2_ levels.

Post-genotyping quality control for all four studies was as described in (Spurdle *et al*. 2011) and (Painter *et al*. 2015). Individuals with <85% estimated European ancestry based on Identity-By-State (IBS) scores between study individuals and individuals in HapMap (http://hapmap.ncbi.nlm.nih.gov/) were excluded.

### E_2_ datasets

#### The EPIC Norfolk study

Sex-hormone levels, including E_2_ and T concentrations, were measured on subsets of the ∼25 000 participants in the European Prospective Investigation of Cancer-Norfolk cohort study (see Day *et al*. (1999) for details). After recruitment, participants were invited to attend a first health check (HC1), at which a blood sample was taken. A second blood sample was taken from participants who attended the second health check (HC2), ∼3 years after the first. For each set of blood samples, a subset were randomly selected for hormone level measurement from among the women who were considered to be post-menopausal based on being >55 years, not having menstruated in the last year, and having not taken HRT for at least 3 months before sampling (Supplementary Table 1C, see section on supplementary data given at the end of this article). The plasma and serum samples collected from these women had been stored at −70 °C until analysis, and their whole blood samples had been stored at −30 °C before DNA extraction. 2368 of the women for whom hormone levels had been measured had also been genotyped using the iCOGS array as BCAC control subjects (and thus were also controls in our iCOGS endometrial cancer analysis). Of these, 1333 women had hormones measured from their HC1 blood sample and 1536 from their HC2 sample, of whom 501 had hormones measured from both samples. Where two measurements existed, we chose the measurement from HC2, when women would be further from the menopause. After excluding women within 2 years of the menopause at blood draw or with missing E_2_ levels or E_2_ values >300 pmol/l (i.e., outside the possible range for a post-menopausal woman) our analysis was based on 1500 genotyped women with HC2 E_2_ levels and 425 women with HC1 levels. Of the HC2 women, 1431 also had a valid T measurement (T was not measured as part of HC1).

Ethical approval was obtained from the Norwich Local Research Ethics Committee, LREC 98CN01. All study participants provided written informed consent.

#### The SIBS study

Participants in the Sisters in Breast Screening study (SIBS) were identified through the National Health Service breast screening program in the UK (Prescott *et al*. 2012). A subset of 905 SIBS women who were aged over 55 years at recruitment, 2 or more years since their last menstrual period, and not currently using HRT at the time of blood collection were selected for hormone measurement (Prescott *et al*. 2012). After excluding women with missing or extreme E_2_ levels (as above), 889 women were left, of whom 302 had been genotyped using the iCOGS array as BCAC control subjects (after quality control exclusions), and thus were also controls in our iCOGS endometrial cancer analysis (Supplementary Table 1C, see section on supplementary data given at the end of this article). All participants gave informed written consent. This study was approved by the Eastern Multicentre Research Ethics Committee (SIBS).

For the EPIC and SIBS studies, plasma E_2_ concentrations were measured at The Royal Marsden Hospital (London, UK), using an in-house RIA using a highly specific rabbit antiserum which had been raised against an E_2_-6-carboxymethyloxime- BSA conjugate and E_2_-6-carboxymethyloxime-[2^−^^125^I] iodohistamine (Dowsett *et al*. 1987). The detection limit was 3 pmol/l and values were replaced with this limit when they were reported as being undetectable. For the SIBS study, at a concentration of 25 pmol/l the within assay variation was 6.5% and the between assay variation was 16% (*n*=18). For the EPIC studies, at a concentration of 18 pmol/l the within assay and the between-batch coefficients of variation (CV) were 8.6 and 13% respectively.

Testosterone was measured in the EPIC and SIBS studies using a solid-phase RIA kit (Diagnostic Products, Gwynedd, UK), with within- and between-batch CV at a concentration of 3.1 nmol/l of 6.1 and 10% respectively and with a detection limit of 0.14 nmol/l.

#### Additional SIBS replication set

To increase statistical power we genotyped all of the 889 SIBS women with E_2_ measurements (described above) for rs727479 using a Custom Taqman Assay (Life Technologies, ThermoFisher Scientific, Waltham, MA, USA) according to manufacturer's instructions (details provided in Supplementary Table 5, see section on supplementary data given at the end of this article). After quality control exclusions described below, 813 women had measured E_2_ measurements and genotypes for rs727479, of whom 264 also had valid iCOGS genotyping i.e., 549 additional samples.

### Regional imputation

We used IMPUTEv2 (Howie *et al*. 2009) to impute genotypes for SNPs in the 50 899 000–52 095 000 region of chromosome 15 in the 1000 Genomes dataset v3 (April 2012 release). We allowed the IMPUTE Software to select the most appropriate haplotypes from among the complete set of 1000 Genomes haplotypes (Howie *et al*. 2011). Imputation was conducted separately for the four datasets, and SNPs with imputation information score <0.7 and/or MAF <0.01 in any of the four studies were excluded.

### Statistical analysis

The four imputed endometrial cancer datasets were analyzed separately using unconditional logistic regression with a per-allele (1 degree of freedom) model using SNPTEST v2 (Ferreira & Marchini 2011). For the iCOGS dataset, analyses were performed adjusting for country and for the first ten principal components, as in (Painter *et al*. 2015). The GWAS datasets were each analyzed as a single stratum, with adjustment for the first two (ANECS and NSECG) and three (SEARCH) principal components. Our ongoing genome-wide analyses have shown that the inclusion of these principal components is sufficient to control for population stratification (genomic control *λ*=1.002–1.038).

The endometrial cancer odds ratios (OR) for the four studies were combined using standard fixed-effects meta-analyses. The *I*^2^ statistic (Higgins & Thompson 2002) was used to estimate the proportion of the variance due to between-study heterogeneity. SNPs with significant between-study heterogeneity (*P*<0.05) were excluded. Analyses for all SNPs were repeated adjusting for the most significant SNP to assess whether multiple independent causal variants were present. A statistical significance cut-off of *P*≤10^−^^4^ was used for secondary and conditional analyses. To determine the most likely candidate causative SNPs, the log likelihoods of all tested SNPs were compared with that of the top SNP. SNPs with log-likelihood ratios of <1:100 of being the top SNP and which were correlated with the top SNP (linkage disequilibrium (LD) *r*^2^>0.2) were prioritized as potentially causal variants for follow-up in bioinformatic and functional analyses (Udler *et al*. 2010).

The analyses were repeated restricting the iCOGS and NSECG studies to those cases with endometrioid or non-endometrioid histology (the ANECS and SEARCH GWAS sample sets contained only endometrioid histology cases).

Associations between SNPs and E_2_ concentrations were tested using the natural logarithm transformed ratio of E_2_ to T concentrations, adjusting for laboratory batch, study (EPIC HC2 or SIBS), age and BMI at blood draw, prior HRT use (yes or no) and menopausal status (2–5 years since menopause or >5 years since menopause) using ProbABEL software (Aulchenko *et al*. 2010). Given the family-based design of the SIBS study, we used the matrix of kinship coefficients to adjust for the non-independence of relatives. This approach is also expected to avoid the effects of population stratification (Chen & Abecasis 2007).

For the analysis including the additional genotyping in the SIBS samples, the data were re-analyzed for all 2767 women, using the sandwich variance estimator to obtain standard errors robust to familial clustering (in the absence of a kinship coefficient matrix for the complete set).

The associations between the most significant SNP and the two phenotypes (endometrial cancer and E_2_ concentration) were repeated after stratifying the datasets according to quartiles of age diagnosis (cases) or interview (controls) or quartiles of BMI. These analyses were restricted to the iCOGS dataset (plus the SIBS replication set for the E_2_ analysis), as BMI was not available for all cases and controls in the GWAS sets. Since T concentration had not been measured in the EPIC HC1 women, the analyses stratified by BMI and age were based on log-transformed E_2_ concentrations uncorrected for T, in order to maximise the sample size and hence the statistical power. Quartiles were based on the variables' distributions in cases, to ensure roughly equivalent statistical power across the quartiles. The same categories were used for the E_2_ analysis to allow direct comparisons between the two phenotypes.

We used a Mendelian randomization style approach to compare the observed association of the top SNP with endometrial cancer with that predicted based on a SNP's effect on E_2_ levels. For this we re-estimated the effect of each effect allele on E_2_ (*β*) adjusting only for study and laboratory batch. Using a published estimate of the endometrial cancer OR associated with a doubling of post-menopausal E_2_ concentration (Lukanova *et al*. 2004), we multiplied the natural logarithm of this OR by the ratio (lnβ/ln2) to obtain a predicted endometrial cancer OR per effect allele. We then compared this predicted OR to that observed, to assess whether the observed association between the SNP and endometrial cancer is compatible with a causal association between higher post-menopausal E_2_ concentration and endometrial cancer. In the same way, we compared the predicted effect of the top SNP on breast cancer risk (based on a published estimate of the effect of doubling E_2_ concentration on breast cancer risk (Key *et al*. 2002)) with that observed in the iCOGS BCAC study of 45 290 breast cancer cases and 41 880 controls of European ancestry (Michailidou *et al*. 2013).

All statistical analyses used R software unless otherwise stated, and all statistical tests were two-sided. The association plot was produced using LocusZoom (Pruim *et al*. 2010).

## Results

### The CYP19A1 association with endometrial cancer is explained by a single signal

Genetic imputation of the ∼1.2 Mb region of chromosome 15 between 50 899 000–52 095 000 using the April 2012 release of the 1000 Genomes reference panel in four independent case–control sets yielded post-QC genotype information for 2937 SNPs in 6608 endometrial cancer cases and 37 925 controls (Supplementary Table 1A, see section on supplementary data given at the end of this article). Of these SNPs, 100 had been genotyped in the largest study (iCOGS), and 191, 201, and 187 in the three GWAS sets (SEARCH, ANECS, and NSECG GWASs respectively).

**Figure 2:**
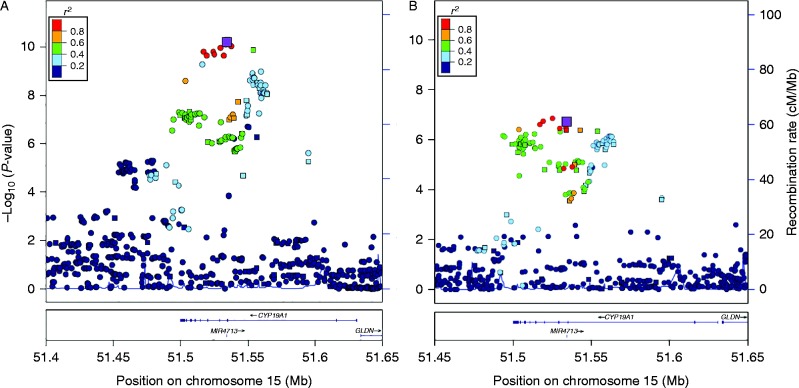
Association of SNPs in the CYP19A1 region with (A) endometrial cancer and (B) E_2_:T, highlighting rs727479. Each point indicates the statistical significance of the association between a SNP and endometrial cancer (Fig. 2A) or between a SNP and the E_2_:T ratio (Fig. 2B). Squares denote SNPs directly genotyped by the iCOGS array; circles are SNPs for which genotypes were imputed. The larger purple square is rs727479, the SNP with the strongest evidence of association with endometrial cancer. Other colours show the strength of linkage disequilibrium between each SNP with rs7277479.

Combining results across the four studies, 171 SNPs had *P*<1×10^−4^, compared with an expected number of less than one under the null hypothesis (Supplementary Table 2, see section on supplementary data given at the end of this article). Fifty SNPs were significant at the conventional GWAS threshold of 5×10^−8^, of which rs727479 in intron 2 was the most significantly associated (OR per A allele=1.15, CI=1.11–1.21, *P*=4.81×10^−11^, Table 1, Fig. 2A). This SNP was directly genotyped in all four studies, and the strength of the association did not differ among studies (*I*^2^=0.0%, *P*_het_=0.92). (Supplementary Table 2, see section on supplementary data given at the end of this article, Supplementary Fig. 1A). Conditioning on rs727479, no other SNPs reached *P*<10^−4^ (Supplementary Table 2).

These results suggest that rs727479, or a SNP correlated with it, is causally related to disease. Based on a likelihood ratio threshold of 1:100 (Udler *et al*. 2010), 28 SNPs remain as possible causal variants (Supplementary Table 2, see section on supplementary data given at the end of this article); all are correlated with rs727479 at *r*^2^>0.2, and five were genotyped in iCOGS (rs7175531, rs727479, rs17601876, rs12050767, and rs749292).

Considering only the 5611 endometrioid–histology cases, 41 SNPs had *P*<5×10^−8^, of which rs727479 was the most significant (OR per A allele=1.16, CI=1.11–1.22, *P*=1.12×10^−10^, Table 1). The effect estimate was somewhat smaller in the analysis restricted to the 887 non-endometrioid cases (OR=1.08, CI=0.98–1.20, *P*=0.13), although the difference in allele frequencies between endometrioid and non-endometrioid cases was not significant (*P*=0.15, Table 1). No SNPs reached *P*<1×10^−4^ in the analysis restricted to the non-endometrioid histology cases (*n*=887) (Supplementary Table 2, see section on supplementary data given at the end of this article).

### Stronger associations between rs727479 and endometrial cancer in women of older age and higher BMI

There was some suggestion of a stronger association between rs727479 and endometrial cancer among older women (OR=1.28 (1.15–1.44) *P*=1.7×10^−5^ and OR=1.24 (1.08–1.42) *P*=1.9×10^−3^ for the third and fourth quartiles of age respectively; Table 1), although the interaction between rs727479 and age was not significant (*P*=0.19).

**Figure 3:**
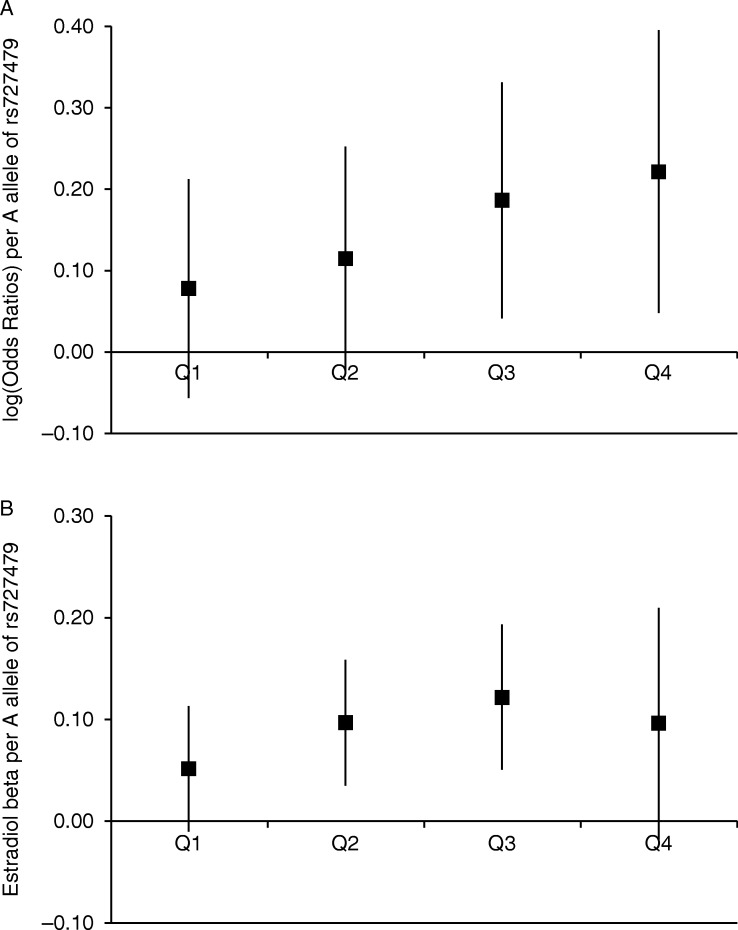
Association of SNP rs727479 with (A) endometrial cancer and (B) E_2_ levels, by quartile of BMI distribution. In Fig. 3A the log(OR) of endometrial cancer associated with each A allele of SNP rs727479 is shown for each quartile of the BMI distribution, adjusting for age. There is a borderline significant interaction between genotype and BMI quartile (*P*=0.047). Figure 3B shows the regression coefficient (*β*) for the association between each A allele of rs727479 and log-transformed E_2_ levels (adjusted for laboratory batch, study, age at blood draw, BMI, HRT use and menopausal status), (*P*_interaction_=0.066). For both plots, the error bars are 95% CI, and the quartiles are based on the BMI distribution in endometrial cancer cases, to allow for comparability between plots, and to ensure sufficient cases in each quartile.

BMI was available for 2858 cases and 14 098 controls from the iCOGS studies. As expected, BMI was positively associated with endometrial cancer risk (OR=1.60 (1.54–1.66) per quartile, *P*<10^−100^). SNP rs727479 was not associated with BMI in previous analyses (*P*=0.94 in the >230 000 GIANT consortium participants (Locke *et al*. 2015))*_ENREF_24* nor in the iCOGS controls (*P*=0.31). There was evidence of a stronger disease association for rs727479 among women with higher BMI (interaction *P*=0.034, which was slightly attenuated when adjusted for age, *P*=0.047), with the strongest association among women in the highest quartile (OR=1.25 (1.05–1.49), *P*=0.012, adjusting for age), (Table 1, Fig. 3A).

### The set of correlated SNPs most significantly associated with endometrial cancer are all within the set of SNPs most significantly associated with the E_2_:T ratio

T is the substrate for aromatization to E_2_, and the ratio of E_2_ to T concentrations, in essence, corrects for the variation in T levels. This correction would be expected to lead to a more direct relationship with aromatase activity, hence we used the E_2_:T ratio as the hormonal phenotype in our initial fine-mapping of the *CYP19A1* region. Circulating E_2_ and T concentrations were measured in 1733 healthy post-menopausal women from the EPIC Norfolk (*N*=1431) and SIBS (*N*=302) studies (Dunning *et al*. 2004, Prescott *et al*. 2012) who formed a subset of the controls in the iCOGS study. Imputation and post-imputation QC identical to that performed in the endometrial cancer analysis resulted in 1956 SNPs across the *CYP19A1* region, of which 100 had been genotyped.

Adjusting for age, BMI, HRT use and menopausal status, 105 SNPs were associated with the E_2_:T ratio at *P*<1×10^−4^, including the lead endometrial cancer SNP rs727479 (*P*=2.06×10^−7^). Two imputed SNPs had very slightly smaller *P* values than rs727479 (rs12592697, *P*=1.46×10^−7^ and rs4775935, *P*=1.89×10^−7^), both of which were in near-complete LD with rs727479 (*r*^2^=0.99). Ninety four SNPs had odds of at least 1:100 compared with rs12592697 of being the causal E_2_:T SNP, and also have *r*^2^>0.2 with rs12592697 (Supplementary Table 2, see section on supplementary data given at the end of this article). Conditioning on rs12592697, no SNPs have *P*<1×10^−4^; hence there is no evidence of a second signal for E_2_:T in this region. The set of 95 SNPs contains all 28 non-excluded endometrial cancer candidate SNPs. Since rs727479 was the most significant of the genotyped SNPs and was statistically almost indistinguishable from the top two SNPs, rs727479 was used as the representative SNP for the set of 95 non-excluded SNPs. The rs727479 A allele was associated with higher E_2_ concentration (*β*=0.092, *P*=3.80×10^−5^) and, to a lesser extent, with lower T concentration (*β*=−0.045, *P*=0.057).

Including an additional 485 women from the EPIC-Norfolk cohort for whom E_2_ but not T concentrations had been measured (and therefore the E_2_:T ratio could not be computed), the association between rs727479 and E_2_ became stronger (*β*=0.094, *P*=3.1×10^−6^). To further increase the statistical power we genotyped rs727479 in the remaining 549 SIBS samples for whom E_2_ concentrations had been measured. In the full set of 2767 women, the association between E_2_ concentrations and rs727479 approached the genome-wide significance threshold (*β*=0.096, *P*=7.4×10^−8^) (Table 1).

There was no evidence of a difference in the association between rs727479 and E_2_ concentration with age (*P*_interaction_=0.90, Table 1). The rs727479-E2 association was the strongest among women with the highest BMIs, with borderline significant evidence of an interaction (*P*_interaction_=0.066, Table 1, Fig. 3B).

### Evidence that higher E_2_ concentration is causal for endometrial cancer

**Figure 4:**
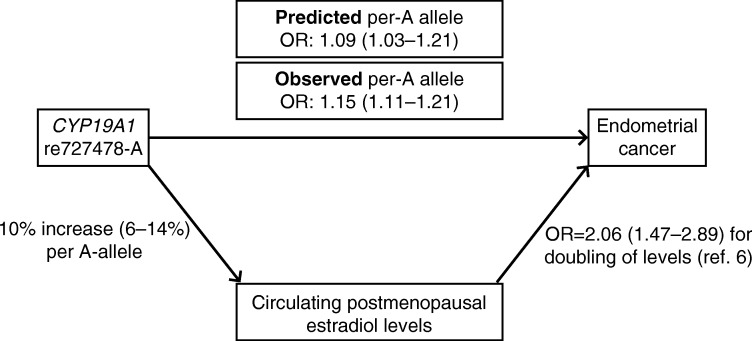
The observed and predicted risks of endometrial cancer associated with each rs727479 A allele. The Observed per-A allele OR is that observed in this study of 6608 and 37 925 endometrial cancer cases and controls. The predicted per-A allele OR is estimated based on the observed association between rs727479 and E_2_ levels in 2767 healthy post-menopausal women, and on the endometrial cancer OR associated with a doubling of post-menopausal E_2_ levels reported by Lukanova *et al*. (2004).

Following a Mendelian randomization argument, if elevated E_2_ concentration were causally associated with endometrial cancer (as opposed to an association produced by confounding), then we would expect any SNP which raises E_2_ to be proportionally associated with endometrial cancer. We observed an rs727429 per-A-allele increase in adjusted E_2_ concentration of 10% (95% CI=6–14%, from the regression coefficient in Table 1 for log-transformed levels). Lukanova *et al*. (2004) estimated that the odds ratio for endometrial cancer associated with a doubling of post-menopausal E_2_ concentration was 2.06 (CI=1.47–2.89; it was necessary to use an external estimate because hormone levels had only been measured in control subjects in our study). Based on this published estimate, the predicted per-allele OR for endometrial cancer would be 1.09 (CI=1.03–1.21), which is consistent with that observed in our study (OR=1.15, CI=1.11–1.21) (Fig. 4).

### Candidate causal variants may regulate CYP19 expression

**Figure 5:**
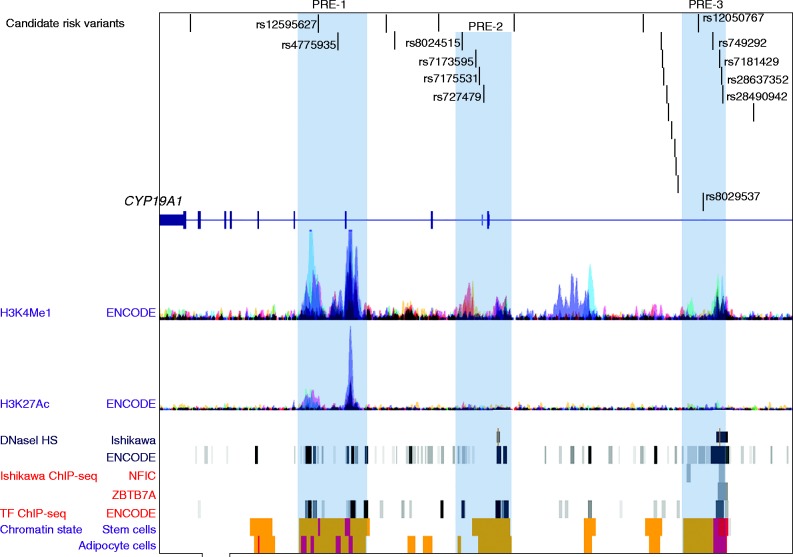
Candidate endometrial risk variants coincide with three PREs. The 28 best candidate causal SNPs map towards the 3′ end of the *CYP19A1* gene. The functional elements displayed were accessed through the UCSC Genome Browser and include: H3K4Me1 and H3K27Ac histone modifications measured by the Encyclopedia of DNA Elements (ENCODE) project in seven cell lines; open chromatin as delineated by DNaseI hypersensitivity sites (HS) in Ishikawa endometrial cancer cells (previously incorrectly named ECC-1)) and 125 other cell types; TF binding in Ishikawa cells and 91 cell lines within ENCODE: 21/28 candidates are predicted to overlap TF binding sites. Roadmap Epigenomics Project chromatin state segmentation of adipose-derived mesenchymal stem cells and adipocytes: orange bars represent enhancers and red bars represent regions flanking active transcription start sites. Twelve SNPs, marked by dbSNP rsIDs, are located in PREs: highlighted in blue. PREs were defined by the presence of histone modifications, DHS, TF binding and Roadmap enhancers.

Bioinformatic analysis defined three putative regulatory elements (PREs) coincident with 12 of 28 candidate endometrial cancer causal variants prioritized by genetic analysis (Fig. 5). Altered binding of transcription factors was predicted for 10/12 candidates located within PREs, including top candidate rs727479 (Supplementary Table 3, see section on supplementary data given at the end of this article). For four of these (rs8024515, rs7181429, rs28637352, and rs28490942) there was experimental evidence for differential transcription factor (TF) binding in the cell types tested by ENCODE (Fig. 5) and SNPs rs7181429, rs28637352 overlap binding consensus sequences for NFIC and ZBTB7A in Ishikawa endometrial cancer cells (Fig. 5). Expression analysis identified nominal associations (*P*<0.05) between risk alleles for the 28 candidate causal variants and greater *CYP19A1* expression in several tissues, with candidate SNP rs7181429's association with expression in blood passing a Bonferroni-corrected significance threshold (*P*=6.0×10^−5^; corrected *P*=1.62×10^−4^; Supplementary Table 4, see section on supplementary data given at the end of this article).

## Discussion

We conducted the largest comprehensive genetic study to date of SNPs across the *CYP19A1* hormone metabolism gene and their associations with both endometrial cancer risk and circulating E_2_ concentration. Using genotype information on nearly 3000 SNPs we have, for the first time, identified GWAS-level significant associations between SNPs in this region and endometrial cancer. Our finding that rs727479 is the most significantly associated SNP in this region confirms the findings of a previous candidate-SNP study (Setiawan *et al*. 2009) and provides a list of 28 SNPs which cannot be excluded as causal on the basis of statistical analyses. We found no evidence for further causal variants outside of this set. For example, rs749292, previously reported as a possible second signal (Setiawan *et al*. 2009), was not significantly associated with risk in our analysis after conditioning on rs727479, given the number of SNPs included in the analysis (*P*_cond_=0.017).

This is the first study to look at the *CYP19A1* endometrial cancer association by histology. The most significant risk SNP, rs727479, appears to be more strongly associated with endometrioid histology endometrial tumors than with the rarer and poorer prognosis non-endometrioid cancers. However, the confidence intervals for the two ORs are not incompatible, and there was no significant difference in allele frequencies between the women with endometrioid and non-endometrioid tumors (*P*=0.15). Despite the common description of non-endometrioid tumours as ‘estrogen independent’, recent work has shown that the two subtypes largely share common risk factors, including those factors relating to endogenous or exogenous estrogen exposure (Setiawan *et al*. 2013), consistent with our findings.

We also confirmed at a borderline GWAS significance level (*P*=7.4×10^−8^) the association between rs727479 and E_2_ concentration previously reported in post-menopausal women (Haiman *et al*. 2007, Ahn *et al*. 2009, Beckmann *et al*. 2011, Prescott *et al*. 2012) and in males (Travis *et al*. 2009). It had been reported that rs749292 and rs727479 may act independently to alter levels (Haiman *et al*. 2007), but we found no association between rs749292 and E_2_ concentration after conditioning on rs727479 (*P*=0.20). Our sample set partially overlaps with that included in the GWAS of hormone levels reported by Prescott *et al*. (2012), (the additional 549 SIBS women genotyped here for rs727479 had all been included in the Prescott *et al*. GWAS), but a combination of nearly 2000 extra subjects, denser genotyping in and around the *CYP19A1* gene and imputation to the 1000 Genomes reference panel allowed us to look in more detail at the region. Our results suggest the existence of a single causal variant in *CYP19A1* underlying both E_2_ concentration and endometrial cancer, although we cannot exclude the possibility that there are instead multiple causal variants which are in sufficiently strong linkage disequilibrium that they are indistinguishable by epidemiological analysis.

We estimate that rs727479 accounts for 1.1% of the variance in post-menopausal E_2_ concentration (in contrast, BMI accounts for 16% of the variance). Given that the estimated heritability of post-menopausal E_2_ is around 40% (Varghese *et al*. 2012) it is clear that further genetic variants that affect E_2_ concentration remain to be found.

The predominant source of circulating estrogens in post-menopausal women is adrenal androgens (T), which are converted to estrogens in peripheral adipose tissues, with the final stage of this process requiring aromatase, the enzyme encoded by the *CYP19A1* gene. Although E_2_ concentrations and endometrial cancer risks are both higher in women with larger BMI regardless of *CYP19A1* genotype, there also appears to be a gene-environment interaction such that the associations of the rs727479 A allele with E_2_ concentration and also with endometrial cancer risk increase according to BMI, with BMI presumably serving as a proxy for the amount of adipose tissue (Fig. 1). Whole body aromatization is known to be directly associated with BMI and the aromatization rate per cell has been found to increase with increasing age (Cleland *et al*. 1985). Together these data suggest that the influence of the SNP may be more profound when the aromatization rate is already higher.

Twelve of the 28 candidate causal variants (including top candidate rs727429) lie in PREs. Further, the risk alleles of candidates located in PRE-3 associate with increased *CYP19A1* expression, and ENCODE and other data (Eeckhoute *et al*. 2006, Lee & Maeda 2012) indicate that the NFIC and ZBTB7A TFs may affect PRE-3 repressor activity in endometrial cancer cells. Taken together these lines of evidence indicate that candidate causal variants within PRE3 should have high priority for follow-up studies to test their effects on *CYP19A1* promoter activity through long-range chromatin interactions.

Using a Mendelian randomization argument with *CYP19A1* genotype as the instrumental variable, we have shown that the endometrial cancer OR per A-allele of rs727479 predicted on the basis of the per-allele effect on E_2_ (1.09, CI=1.03–1.21) is in line with the directly observed effect of each A allele on endometrial cancer (OR=1.15, CI=1.11–1.21) (Fig. 4). Whereas previous epidemiological studies have observed a positive correlation between E_2_ concentration and risk, it has not been possible to distinguish between a causal relationship and one produced by confounding. By exploiting the random allocation of alleles to individuals at conception, Mendelian randomization mimics a randomized control trial, thus removing possible confounding. We have therefore found good evidence that higher post-menopausal E_2_ concentrations are indeed a causal risk factor for endometrial cancer, in line with other evidence such as the observed increase in risk associated with estrogen-only HRT but not with estrogen+progesterone HRT (Beral *et al*. 2005). Hence lowering E_2_ levels has the potential to be a useful strategy for reducing risk. Ideally we would like to be able to test this hypothesis in a prospective study, whereby E_2_ levels are measured at baseline for a cohort of healthy post-menopausal women who are then followed up for endometrial cancer incidence. However, such a study would need to be extremely large in order to accrue sufficient cancer cases within a reasonable time frame. It would also be interesting to repeat the study in a non-European setting in order to see whether the results are consistent across populations.

In Mendelian randomization, for a genetic variant to be a suitable instrument, in addition to being associated with the biomarker it must also be i) independent of the unobserved confounders of the biomarker-disease relationship and ii) associated with the disease only via the biomarker. Population stratification is the most obvious way in which condition i) can be violated. To guard against this we restricted our study to subjects of European ancestry and adjusted for principal components. Condition ii) can be broken by pleiotropy, or similarly if the variant is in LD with a separate disease-associated variant. It is impossible to be certain that this condition has been met, and our finding that the observed association between rs727479 and endometrial cancer is slightly stronger than that predicted according to rs727479's effect on E_2_ levels may in part be due to the SNP additionally acting on endometrial cancer risk via a pathway not involving circulating E_2_. A Mendelian randomization using an instrument consisting of multiple independent E_2_-associated SNPs, as and when they are reported (e.g., via larger GWAS) is one way to minimize the potential impact of pleiotropy on the results. We are confident that the results are not due to reverse causation, since E_2_ measurements were all carried out in women from the control arm of the endometrial cancer study.

Despite higher endogenous E_2_ concentration also being a known risk factor for breast cancer (Key *et al*. 2002), candidate SNP studies of *CYP19A1* have not reported an association with breast cancer (Haiman *et al*. 2007). Based on the 45 290 European-ancestry breast cancer cases and 41 880 controls from the BCAC iCOGS study, none of the 171 *CYP19A1* locus SNPs with *P*<10^−4^ for endometrial cancer were associated with breast cancer (Michailidou *et al*. 2013) (minimum *P*=0.0033, data not shown). This may in part be because E_2_ concentration is less strongly related to breast than to endometrial cancer; a doubling of E_2_ concentration has been reported to be associated with an OR of 1.29 (CI=1.15–1.44) for breast cancer (Key *et al*. 2002), from which we would predict a breast cancer OR of 1.03 (1.01–1.07) per A allele of rs727479. This predicted effect size is consistent with that observed for breast cancer in BCAC (OR=1.02 (1.00–1.04); *P*=0.10), but the effect size is too small to be confidently detected, even in a breast cancer study of this size.

In conclusion, we have confirmed at a genome-wide-level of significance the association between endometrial cancer and variants within the *CYP19A1* gene, and shown that all of the reported associations can be explained by a single risk peak. We have also provided evidence that the same set of variants is associated with higher E_2_ concentration in post-menopausal women, supporting a causal role for E_2_ in endometrial cancer. For both traits, the SNP associations were stronger in women with a higher BMI, suggesting a biologically plausible gene-environment interaction.

## Supplementary data

This is linked to the online version of the paper at http://dx.doi.org/10.1530/ERC-15-0386.

## Author contribution statement

A M Dunning, D F Easton, K-T Khaw, G W Montgomery, P D P Pharoah, A B Spurdle, I Tomlinson, and P M Webb obtained funding for the study. D F Easton and A B Spurdle designed the study and A B Spurdle and D J Thompson drafted the manuscript. D J Thompson conducted all statistical analyses and genotype imputation. D M Glubb and T A O'Mara conducted bioinformatic analyses. T A O'Mara and J N Painter co-ordinated the endometrial cancer iCOGS genotyping, and associated data management. J Dennis, K Michailidou and J P Tyrer co-ordinated quality control and data cleaning for the iCOGS control datasets, and K Michailidou provided quality control for the SEARCH GWAS control set. M K Bolla, Q Wang, M Shah, and R Luben were responsible for data management. T A O'Mara and A B Spurdle co-ordinated the ANECS GWAS genotyping; A M Dunning co-ordinated the SEARCH GWAS genotyping; I Tomlinson co-ordinated the NSECG GWAS genotyping. E Folkerd, D Doody, and M Dürst carried out the hormone measurements. The remaining authors were involved in the co-ordination and/or extraction of phenotypic information for contributing studies. All authors provided critical review of the manuscript.

## Figures and Tables

**Table 1:** SNP rs727479 A-allele associations with endometrial cancer and with circulating E_2_ levels

	**N_cases_/N_ctrls_**	**f_case_/f_ctrl_**	**OR** (95% CI)	***P* value**	***P*_interaction_**
Endometrial cancer	6608/37 925	0.688/0.651	1.15 (1.11, 1.21)	4.81×10^−11^	
BMI Q1 (<24.3 kg/m^2^)	715/5463	0.668/0.643	1.08 (0.95, 1.24)	0.25	
BMI Q2 (24.3–28.0 kg/m^2^)	718/4372	0.677/0.648	1.12 (0.98, 1.29)	0.10	
BMI Q3 (28.0–33.2 kg/m^2^)	702/2910	0.697/0.648	1.21 (1.04, 1.40)	0.012	
BMI Q4 (≥33.2 kg/m^2^)	721/1254	0.709/0.651	1.25 (1.05, 1.49)	0.012	0.047
Age Q1 (<57.0 years)	1005/14 106	0.679/0.653	1.10 (1.00, 1.22)	0.060	
Age Q2 (57.0–63.0years)	1079/5309	0.679/0.652	1.10 (0.98, 1.23)	0.10	
Age Q3 (63.0–69.0 years)	1145/4699	0.693/0.644	1.28 (1.15, 1.44)	1.73×10^−5^	
Age Q4 (≥69.0 years)	1081/2948	0.701/0.651	1.24 (1.08, 1.42)	0.0019	0.19
Histology:					
Endometrioid	5611/37 925	0.690/0.651	1.16 (1.11, 1.22)	1.12×10^−10^	
Non-endometrioid	887/37 925	0.678/0.651	1.08 (0.98, 1.20)	0.13	0.15
	***N***_**samples**_	***f***	***β* (se)**	***P* value**	
E_2_ levels	2767	0.656	0.096 (0.018)	7.40×10^−8^	
BMI Q1 (<24.3 kg/m^2^)	868	0.656	0.052 (0.032)	0.11	
BMI Q2 (24.3–28.0 kg/m^2^)	985	0.650	0.097 (0.031)	0.0020	
BMI Q3 (28.0–33.2 kg/m^2^)	664	0.671	0.122 (0.036)	8.45×10^−4^	
BMI Q4 (≥33.2 kg/m^2^)	250	0.642	0.096 (0.058)	0.099	0.066
Age Q1 (<57.0 years)	287	0.656	0.095 (0.083)	0.25	
Age Q2 (57.0–63.0 years)	802	0.661	0.104 (0.030)	5.65×10^−4^	
Age Q3 (63.0–69.0 years)	789	0.653	0.080 (0.032)	0.013	
Age Q4 (≥69.0 years)	889	0.654	0.105 (0.031)	6.48×10^−4^	0.90

*F*, frequency of the rs727479 A allele; age is in years; Q1–Q4 are quartiles of the distribution of BMI or age in the endometrial cases. The endometrial cancer analysis by quartiles is adjusted for age. E_2_ concentrations are log transformed and adjusted for laboratory batch, study, age at blood draw, BMI, HRT use and menopausal status.
